# Supplementary material for: Surface Electromyography-Driven Parameters for Representing Muscle Mass and Strength
Source: Sensors (Basel). 2023 Jun 10;23(12):5490. doi: 10.3390/s23125490 (PMC10302141; doi:10.3390/s23125490)
Supplement: Supplementary file 1 [file sensors-23-05490-s001.zip › sensors-2388900-supplementary.pdf]

Supplementary Table S1. Stepwise regression analysis for ASM incorporating upper limb related sEMG variables (Model 1) and lower limb related sEMG variables (Model 2).

<Model 1>

Entered predictor variables	Equation: ASM=−27.590+21.471×Height+0.172×weight−2.093× (1, if,female; 0, if male)+0.361×RatioRMS(KF)+0.405×RatioRMS(KE)				
		β	Standard error	VIF	*P*-value
Age, sex, height, weight, MeanRMS(KF, KE), MaxRMS(KF, KE), RatioRMS(KF, KE)	Constant	−27.590	3.213		
	Height (m)	21.471	2.044	3.082	<0.001
	Weight (kg)	0.172	0.013	1.928	<0.001
	Sex (female)	−2.093	0.340	2.812	<0.001
	RatioRMS(KF) (mV)	0.361	0.118	1.251	0.003
	RatioRMS(KE) (mV)	0.405	0.180	1.055	0.026
	R^2	0.936			
	Adjusted R^2	0.934			
	SEE	1.174			
	Durbin-Watson statistic	1.703			

<Model 2>

Entered predictor variables	Equation: ASM=−25.584+20.775×Height+0.174×weight−2.297 (1, if,female; 0, if male)+1.109×MeanRMS(EE)				
		β	Standard error	VIF	*P*-value
Age, sex, height, weight, MeanRMS(EF, EE), MaxRMS(EF, EE), RatioRMS(EF, EE)	Constant	−25.584	3.230		
	Height (m)	20.775	2.103	3.144	<0.001
	Weight (kg)	0.174	0.013	1.953	<0.001
	Sex (female)	−2.297	0.336	2.651	<0.001
	MeanRMS(EE) (mV)	1.109	0.378	1.245	0.004
	R^2	0.933			
	Adjusted R^2	0.931			
	SEE	1.196			
	Durbin-Watson statistic	1.934			

ASM, appendicular skeletal muscle mass; sEMG, surface electromyography; RMS, root mean

square; EF, elbow flexion; EE, elbow extension; KF, knee flexion; KE, knee extension; SEE, standard error of estimate; VIF, variance inflation factor.
